# Death of Dr. Mendenhall

**Published:** 1874-07

**Authors:** 


### DEATH OF DR. MENDENHALL.

Dr. Geo. Mendenhall, of this city, died at 10 p. m., June 4th, in his residence on Eighth st., after a lingering illness of nearly ten months.

For some months prior to the fall of 1872, he felt the necessity of relaxing his rigid business habits, and by the urgent advice of his medical friends sought recuperation for his wasted energies by a trip to Europe, but in August last, only a few months after his return home, he was smitten with paralysis and never recovered.

He passed calmly away, surrounded by his devoted family, and will be sincerely mourned by a very large circle of friends, to whom he was endeared by a thousand acts of unostentatious kindness, growing out of a long and honorable professional career in the city of his adoption.

We are able to present the following authentic account of his life:

Dr. George Mendenhall was the fourth son of Aaron and Lydia Mendenhall, and was born in the village of Sharon, Beaver county, Pennsylvania, May 5th, 1814. He was descended from the early Quaker settlers of Pennsylvania, one of his paternal ancestors having taken a prominent part with William Penn in the "Elm Tree Treaty" with the Indians. His mother was a sister of the Hon. Joseph Richardson, Speaker of the House of Representatives of Ohio, from 1818 to 1822. During his early childhood his parents removed to the vicinity of Fairfield, Columbiana county, Ohio, where he spent the greater part of his minority and received such education as the schools of the neighborhood offered in addition to which his father, who was a man of learning and cultivation, with a good library, imparted to him much valuable instruction. He was a lad of studious and industrious habits, but lost the valuable assistance of his father at the age of fourteen. His health at that time was delicate, which prevented continuance in the employment of a country store. While in this employment he devoted spare time to reading books on chemistry and pharmacy, suggested by his duties in dealing out drugs and medicines to the customers of the store. In this he was encouraged by his brother and employers, who also suggested the regular sudy of medicine. As a prerequisite he mastered Latin, and entered the office of the late Dr. Ben jamin Stanton, of Salem, Ohio, as a student. Dr. Stanton was an uncle to the late Hon. E. M. Stanton, and a man in every way qualified to impart instruction to his young student. His next step was to enter the University of Pennsylvania, where he received instruction under Drs. Chapman, Jackson, Ditson, Korner, Hare and Dewess, and graduated with credit in March, 1835, being then about twenty-one years of age. In the ensuing year he opened an office in Cleveland, Ohio, at that time a city of only five thousand inhabitants. He won his way with the profession and obtained a practice. In the autumn of 1837 he again went to Philadelphia, then the recognized emporium of medical science in America, and received the appointment of resident physician to the Philadelphia Hospital, and remained until the ensuing spring, when he was attacked by a malignant form of petchial typhus, then prevalent in the hospital. By the assiduous attention of the consulting physicians, Drs. Pennock and Gerhard, he was enabled to leave the hospital alive.

"In October 1838 Dr. Mendenhall was united in marriage to Miss Elizabeth S. Maule, of Philadelphia, formerly of Richmond, Virginia. He returned to Cleveland and resumed the practice of medicine with renewed energy, gradually increasing his business and establishing himself in public favor until January, 1843, when he was attacted by pulmonary disease that threatened permanent loss of health, and compelled him to seek a more genial climate than the region of the lakes afforded.

"Dr. Mendenhall removed to Cincinnati in October, 1843, under the most discouraging circumstances of impaired health, no practice, and a dependent family; but his natural energy and hopeful disposition, and a wife always ready to promote his success by adapting her wants to their means, lightened his burden until by long and patient effort success once more shone upon his path, and brightened his prospects.

Soon after his arrival in Cincinnati he associated himself with Drs. Vattier, Chamberlain, Warder and Williams in the conduct of the City Dispensary, a charitable medical institution supported entirely by private benevolence, and with no compensation to its attending physicians. In company with some of the last named gentlemen and others, Dr. Mendenhall organized a summer school of medicine which was carried on with much success for several years.

In the summer of 1848 he was elected to the professorship of dental pathology and therapeutics in the Ohio College of Dental Surgery. He filled this position to the eminent satisfaction of the trustees and faculty of the college, and the classes whom he taught. Many memories will be revived by this sad intelligence in those who were in his charge as pupils. Such was his bearing, as to command the respect, and secure the respect and esteem of all who knew him.

Through the press of professional occupancy he was compelled to resign his position in the Dental College in Feb. 1853. The chair was subsequentaly occupied by one of his pupils.

In 1852 he engaged with several prominent medical men of the city in organizing the Miami Medical College, an enterprise that was attended with marked success, as its results attest. All through the twenty years dating from that time Dr. Mendenhall larbored with unremitting pains as a practitioner, as a contributor to medical literature, and as a medical teacher. He was characterized by a singular devotion to his profession. His promptness to the bedside of his patients established his character for reliability, a quality absolutely indispensable to the successful physician, and he built around him a solid wall of friends who learned to recognize in him the highest qualities of manhood—great humanity and unflinching patriotism.

He rests in the solemn dignity of death, but his good name will live after him, and add to the honor and respect in which the noble profession is held by all intelligent people.

